# Late onset epilepsy associated with marijuana abuse: a case report with MRI findings

**DOI:** 10.11604/pamj.2014.17.158.2402

**Published:** 2014-03-04

**Authors:** Yannick Fogoum Fogang, Massaman Camara, Paul Chimi Mbonda, Dènahin Toffa, Kamadore Touré

**Affiliations:** 1 Neurology Department, Fann teaching Hospital, Dakar, Senegal

**Keywords:** Symptomatic epilepsy, intracerebral haemorrhage, marijuana, Magnetic Resonance Imaging

## Abstract

Marijuana is the most widely used illicit substance in the world. The relation between marijuana use and epileptic seizures is still controversial. We report a case of late onset epilepsy associated with marijuana abuse, with brain magnetic resonance imaging (MRI) findings. A 44-year-old patient was admitted for 03 isolated episodes of secondary generalized tonic-clonic seizures. He had a history of 26 years regular marijuana smoking. On admission, we found a tachycardia, psychomotor slowing, asymmetric hyperreflexia, bilateral Babinski sign without weakness. Laboratory work-up showed a high level of urine of Δ-9-tétrahydroxycannabinol. Electroencephalogram was normal. Brain MRI revealed abnormal signal intensities in the right frontal lobe and basal ganglia. Seizures cessation was obtained with anti-epileptic treatment. We suggest that marijuana abuse through vascular and toxic mechanisms could explain seizures in this case.

## Introduction

Marijuana is a naturally growing plant, with many chemical constituents. Approximately 60 cannabinoids and 260 non cannabinoid constituents have been identified [1]. Marijuana is the most widely used illicit substance in the world [2]. It is generally smoked, but may also be ingested. Acute administration produces diverse cognitive, perceptual, and cardiovascular effects [3]. The association between marijuana and epileptic seizures is still controversial and clinico-radiological case reports are seldom. Interestingly, some evidence suggests that marijuana and its active cannabinoids have antiepileptic effects, especially for focal or tonic-clonic seizures [4–5]. We describe here a case of late-onset epilepsy associated with marijuana abuse, with magnetic resonance imaging (MRI) correlations.

## Patient and observation

The patient was a 44-year-old man, single and jobless who was admitted in the neurology department for three isolated episodes in five hours, of secondary generalized left body side tonic-clonic seizures lasting less than ten minutes each. He presented 08 months before this admission a severe headache of abrupt onset during a period of heavy smoking of marijuana, associated with one generalized tonic-clonic seizure. He consulted at a Health Dispensary where he was prescribed, without any brain imaging phenobarbital: 100 mg/day and paracetamol for pain, but his compliance to treatment was poor. However, he did not have any seizure until this consultation. He had a history of regular marijuana smoking for 26 years, but no history of recurrent headache or seizures in childhood and in his family. There was no notion of alcohol intake, or head trauma before the onset of symptoms. On admission, one day after the last seizure, his general state was good, vital signs revealed a blood pressure of 110/80 mmHg, a pulse rate of 104 beats per minute, a respiration rate of 18 breaths per minute, a temperature of 37.2°C, a weight of 56 kg and a height of 1.62 m. Neurologic examination revealed an arouse patient, with psychomotor slowing. Pupils were equal and reactive. We found a bilateral and asymmetric hyperreflexia predominant on the left body side, bilateral Babinski sign, but the muscle strength was normal. The cardiac examination revealed a regular tachycardia without murmurs.

Urine analysis showed a high level of Δ-9-tétrahydroxycannabinol (Δ-9-THC), superior to 150 ng/ml. Full blood count, Erythrocyte Sedimentation Rate, C-reactive protein level, fasting blood sugar, serum urea and creatinin levels, liver function test, serum levels of sodium, potassium, calcium and magnesium, HIV and syphilis serologies were all normal. Cerebro-spinal fluid analysis was also normal.

An electrocardiogram was done and showed a sinus tachycardia with a heart rate of 102 beats per minute.

An electroencephalogram performed four days after the last seizure was normal.

**Figure 1:**
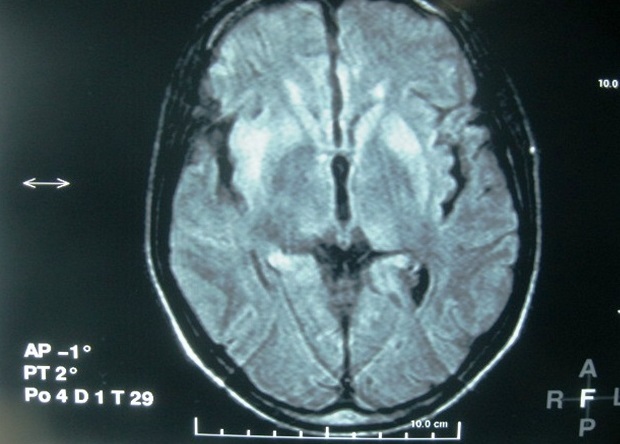
Brain MRI showing focal hyperintensity of the right sub-cortico-frontal region on FLAIR image

**Figure 2:**
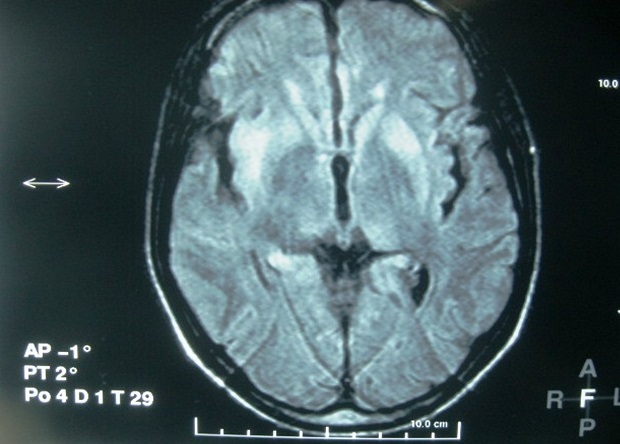
Brain MRI showing bilateral and symmetric hyperintensity of striatum and hyperintensity of the right insular cortex on FLAIR image

**Figure 3:**
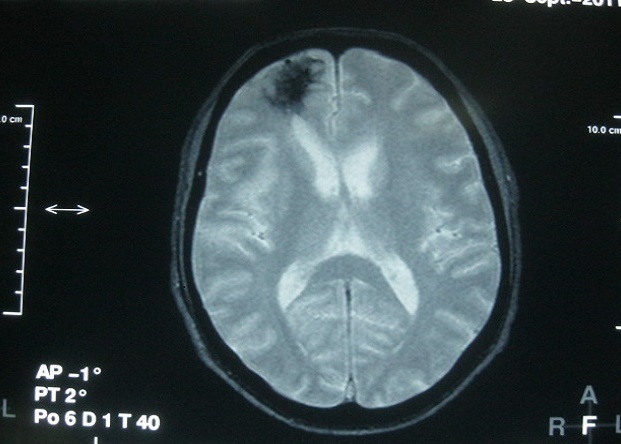
Brain MRI showing hypointensity of the right fronto-polar region on T2^*^ image

A brain MRI on day six after the last seizure revealed on FLAIR images, focal hyperintensity of the right sub-cortico-frontal region (Figure 1) and right insular cortex (Figure 2), bilateral and symmetric hyperintensity of striatum (Figure 2). T2^*^ images showed hypointensity of the right fronto-polar region (Figure 3). Brain MRI angiography did not reveal any vascular malformation.

On admission, patient was boarded on carbamazepine: 200 mg bid, and clobazam: 5mg bid for two weeks. A psychiatric consultation was done for marijuana withdrawal. The patient was discharged after twenty days of admission with carmazepine 200mg bid. After three months of follow-up he did not present any epileptic seizure, and the pulse rate became normal.

## Discussion

Our patient presented with late onset epileptic seizures associated with chronic marijuana use. The duration and frequency of marijuana smoking, the presence of tachycardia, diffuse hyperreflexia and psychomotor slowing are features of drug abuse. The detection of high level of Δ-9-THC in urine indicates marijuana smoking within the last couple of weeks. The asymmetric pattern of hyperreflexia can be attributed to the presence of focal brain lesions. The presence of cortical and subcortical hyperintensities around the right frontal lesion in the peri-ictal period could correspond to transient peri-ictal MRI abnormalities (TPMA). These abnormalities are located around epileptic foci during the peri-ictal period on MRI [6]. Canas and colleagues reported a clinical, electroencephalographic and TPMA concordance in 38.6% of cases [6]. Symmetric and bilateral striatum hyperintensity can be attributed to a toxic effect of marijuana, given the high sensitivity of basal ganglia to toxic and metabolic disturbances. A follow-up brain MRI could have permitted us to follow these abnormalities after marijuana withdrawal, but it was not performed due to economic reasons.

Questions concerning the mechanism of the right frontal lesion and its link with marijuana abuse are addressed. The sudden onset of symptoms is in favour of a vascular mechanism. The T2□ hypointensity in this lesion suggests and old bleeding, either a spontaneous lobar haematoma or hemorrhagic transformation of an ischemic stroke. However, a traumatic brain injury cannot be formally ruled out, even if there is no clinical evidence of head trauma.

Rare cases of hemorrhagic and ischemic strokes attributed to acute use of high doses of marijuana have been described in the literature [7–8]. Chronic marijuana smoking is also considered as a cerebrovascular risk factor [9]. Stroke in marijuana abusers occurs mostly in young adults without other cardiovascular risk factors, who are not taking other drugs, and who have recently increased their use of marijuana [8]. The onset of symptoms during a period of high marijuana consumption, age, and the absence of other cardiovascular risk factors in this case, corresponds to the clinical characteristics of marijuana-induced stroke. The incriminated mechanism of marijuana induced stroke is a toxic cerebral angiopathy with vasospasm, associated with or without hypotension or hypertension [8–9].

## Conclusion

In conclusion, marijuana abuse can lead to various brain abnormalities. We suggest that marijuana abuse, through a combination of vascular and toxic effects on brain could explain epilepsy and other neurological signs in this case. Marijuana should then be used with caution even for therapeutic purposes, given the risk of brain damage.
